# Configuration of ripple domains and their topological defects formed under local mechanical stress on hexagonal monolayer graphene

**DOI:** 10.1038/srep09390

**Published:** 2015-03-24

**Authors:** Yeonggu Park, Jin Sik Choi, Taekjib Choi, Mi Jung Lee, Quanxi Jia, Minwoo Park, Hoonkyung Lee, Bae Ho Park

**Affiliations:** 1 Division of Quantum Phases & devices, Department of Physics, Konkuk University, Seoul, 143-701, Korea; 2 Creative Research Center for Graphene Electronics, Electronics and Telecommunications Research Institute (ETRI), Daejeon 305-700, Korea; 3 Hybrid Materials Research Center, Department of Nanotechnology and Advanced Materials Engineering, Sejong University, Seoul 143-747, Korea; 4 Center for Integrated Nanotechnologies (CINT), Los Alamos National Laboratory, Los Alamos, New Mexico 87545, USA

## Abstract

Ripples in graphene are extensively investigated because they ensure the mechanical stability of two-dimensional graphene and affect its electronic properties. They arise from spontaneous symmetry breaking and are usually manifested in the form of domains with long-range order. It is expected that topological defects accompany a material exhibiting long-range order, whose functionality depends on characteristics of domains and topological defects. However, there remains a lack of understanding regarding ripple domains and their topological defects formed on monolayer graphene. Here we explore configuration of ripple domains and their topological defects in exfoliated monolayer graphenes on SiO_2_/Si substrates using transverse shear microscope. We observe three-color domains with three different ripple directions, which meet at a core. Furthermore, the closed domain is surrounded by an even number of cores connected together by domain boundaries, similar to topological vortex and anti-vortex pairs. In addition, we have found that axisymmetric three-color domains can be induced around nanoparticles underneath the graphene. This fascinating configuration of ripple domains may result from the intrinsic hexagonal symmetry of two-dimensional graphene, which is supported by theoretical simulation using molecular dynamics. Our findings are expected to play a key role in understanding of ripple physics in graphene and other two-dimensional materials.

Two-dimensional crystalline systems and elastic membranes often exhibit spontaneous buckling, bending, rippling, and/or wrinkling. These interesting phenomena arise from the large degree of deformation between bending and stretching caused by external mechanical stimuli, because the elastic energy for stretching or compressing the sheet decreases as a result of out-of-plane deformation^1^,^2^,^3^. Minimization of the total elastic energy by wrinkling of the elastic membrane can accommodate the mechanical instability under applied strain^2^,^3^,^4^,^5^. Since monolayer graphene is a two-dimensional crystalline system with atomic-scale thickness^6^, it exhibits intrinsic rippling with smooth undulations such that out-of-plane elastic deformations can provide stability of graphene in spite of external thermal energy fluctuation^7^,^8^.

The surface corrugations are expected to strongly influence the electronic properties of graphene^9^,^10^,^11^ because local structural deformation is predicted to modify local electron density, which can generate a pseudomagnetic field and change the chemical activity^12^,^13^. It is also suggested that charge conductivity can be limited by ripples where charge carriers are scattered by flexural phonons^14^. In addition, anisotropic behavior is expected for charge propagation due to periodic ripple structures.

Previously, it was reported that ripple domains in exfoliated monolayer graphene on SiO_2_/Si substrate can be observed using contact-mode atomic force microscope (AFM)^15^,^16^. Each ripple domain shows friction anisotropy due to long-range ordering of ripples. This phenomenon may result from inhomogeneous mechanical stress on a two-dimensional hexagonal lattice of graphene during mechanical exfoliation^15^. Recently, we have demonstrated that zigzag directional ripples are preferred in these domains using both AFM and angle-resolved photoemission spectroscopy (ARPES). In addition, we estimated the ripple size as ~5.7 nm by considering the interaction energy between graphene and substrate^17^. This ripple domain with long-range order should be associated with structural spontaneous symmetry breaking that is usually accompanied by topological defects, such as domain boundaries and vortices^18^,^19^,^20^,^21^,^22^. Thus, the exploration of spatial structure of ripple domains is of paramount importance in order to have a better understanding of both elastic deformation and carrier transport of graphene.

Here, we explore configuration of ripple domains as well as related topological defects and their response to stress in exfoliated monolayer graphenes on SiO_2_/Si substrates using transverse shear microscope (TSM), which are difficult to be spatially resolved using transmission electron microscope (TEM) or scanning tunneling microscope (STM). We observe three-color domains with three different ripple directions, which meet at a core. Each closed domain is surrounded by an even number of cores connected together by domain boundaries, similar to topological vortex and anti-vortex pairs. In addition, we have found that axisymmetric three-color domains can be induced around nanoparticles underneath the graphene. This fascinating configuration of ripple domains may result from the intrinsic hexagonal symmetry of two-dimensional graphene, which is supported by theoretical simulation using molecular dynamics. Our findings are expected to play a key role in understanding of ripple physics in graphene and other two-dimensional materials.

## Results and Discussion

**Figure 1:**
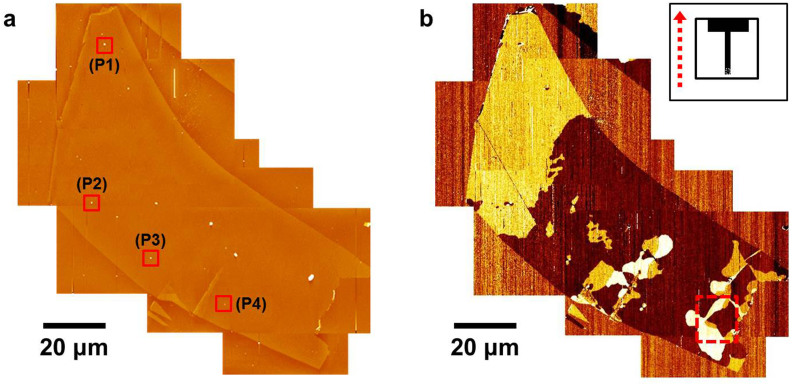
Ripple domains in a single monolayer graphene sheet. (a) AFM topographic image of a single monolayer graphene sheet equivalent to patching 9 partial images with a dimension of 40 × 40 μm^2^, showing folds and wrinkles at the edge. P1, P2, P3, and P4 denote regions with embedded particles under the graphene. (b) TSM image obtained by longitudinal scan across the monolayer graphene sheet. Three domain contrasts correspond to three ripple directions. Red-dashed rectangular area in (b) is described in Figure 2. The inset of (b) shows the top view of the cantilever, and the scan direction is marked by the red-dotted arrow. Note that the prepared graphene sample was treated in high vacuum before imaging.

Monolayer graphenes were prepared on SiO_2_/Si substrate by mechanical exfoliation method and confirmed by Raman spectroscopy (see Supplementary Figure S1 online). During the preparation of graphene samples on SiO_2_/Si substrates, particles were introduced onto the surface of substrates and buried under the graphene layers. Figure 1a shows an AFM topographic image of a single exfoliated monolayer graphene sheet with wrinkled, folded edges and the particles underneath it. The distribution of ripple domains (Figure 1b) is revealed using a TSM, which monitors AFM cantilever torsion resulting from net shear forces on an AFM tip by scanning it in the longitudinal direction with respect to the cantilever body^23^. We demonstrated that ripple domains with different ripple directions could be effectively visualized by measuring the cantilever torsion due to the interaction between the scanning tip and the ripples using TSM and lateral force microscope (LFM)^15^,^16^. Because cantilever torsion measured using TSM can be related with the elastic shear deformation properties of crystal surface^23^, TSM can afford enhanced sensitivity to ripples. Actually, TSM has produced clearer ripple domain contrasts than those produced by LFM^16^.

A single monolayer graphene sheet exhibits complex ripple domain structures that mostly appear near the folded or wrinkled edges. Such local aspects are similar to structural deformation of elastic membrane under an applied stress, where folds induce localized strain that influences wrinkles in their neighborhood^1^,^2^. In other words, the strain localization via folds can lead to regions of increased stress near the folded area. Complex ripple domain structures are often observed in monolayer graphene sheets with wrinkled and folded edges, whereas a wrinkle- or fold-free monolayer graphene sheet only shows a single ripple domain (see Supplementary Figure S2 online)^15^,^16^,^17^. This implies that the strain distribution in graphene under external stress strongly affects the ripple domain structures observed everywhere of graphene. We should note that TSM or LFM signals of each domain fit well with simple sinusoidal functions of sample rotation angle and showed constant line profiles, which might be caused by homogeneously well-arranged ripples in one domain^15^,^16^,^17^. The complex ripple domain structure of our large graphene consists of “only” three-distinctive domains, *i.e.*, there are only three ripple directions in the monolayer graphene, which can be determined by the dependence of TSM and LFM images on the sample rotation angle (see Supplementary Figures S3 and S4 online). Furthermore, we also observed that elastic shear anisotropy (the difference between shear force maximum and minimum values obtained by TSM) and friction anisotropy (by LFM) of each domain were respectively same as those of other domain^15^,^16^. It indicates that each ripple domain has the common origin of such anisotropy and the similar structural features of ripples with those in other domain.

In our previous work (Ref. 17), we have demonstrated that ripples are preferentially formed along the zigzag directions of graphene. Because hexagonal graphene has three zigzag axes, there are only three preferred ripple directions on graphene. Therefore, a tensile (compressive) strain along the direction rotated in the range of −30° to 30° from the positive or negative directions of a zigzag axis (perpendicular-to-zigzag direction) can induce the formation of ripples parallel to the zigzag axis. The area, where is governed by such strains, can be manifested as a domain which has ripples parallel to one zigzag axis. The same color domains, e.g. those around one particle, in a TSM image should have the same directional ripples but may be caused by tensile (compressive) strains along the direction rotated in the range of −30° to 30° from the positive or negative directions of a zigzag axis (perpendicular-to-zigzag direction).

**Figure 2:**
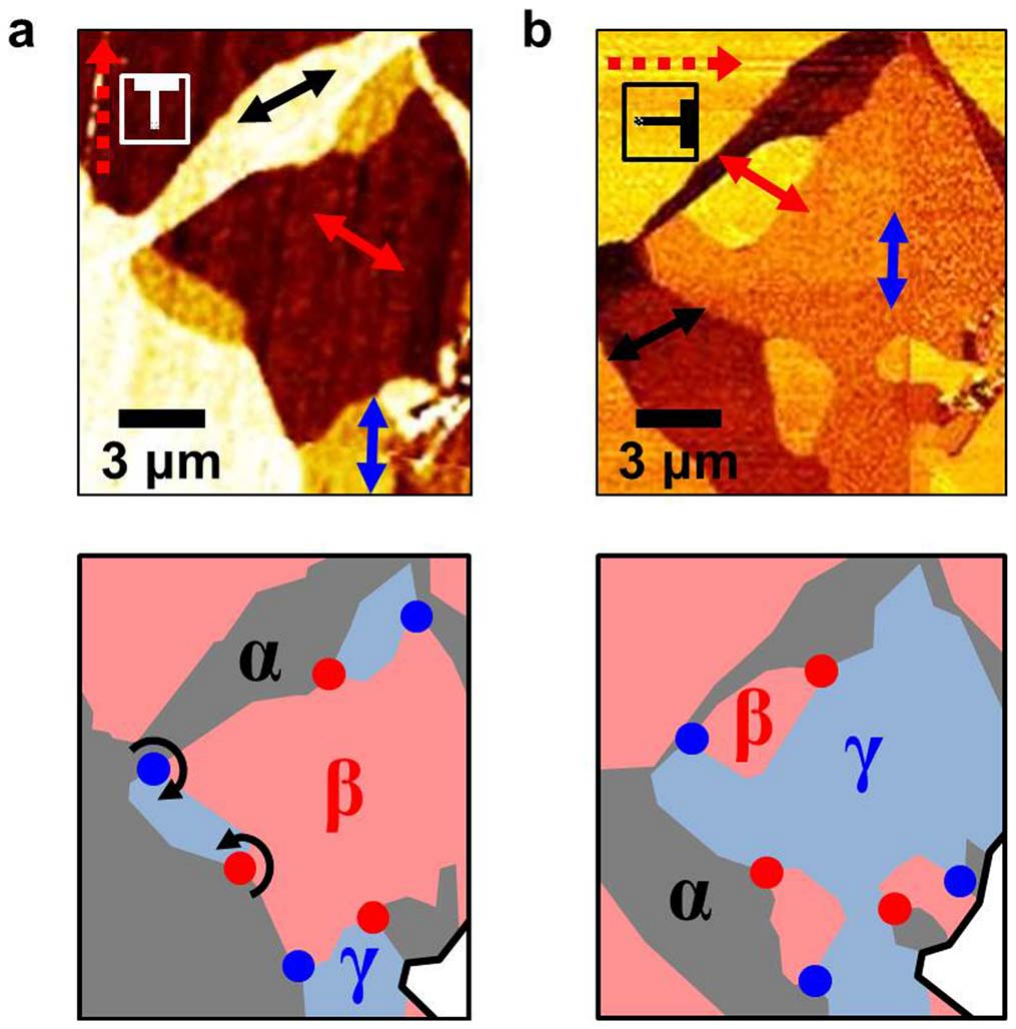
Rearrangement of ripple domain network and accompanied pairing of the vortex and anti-vortex. (a, b) Magnified TSM images of a red-dashed rectangular area in Figure 1b, obtained before (a) and after (b) high humidity exposure (80–90% RH). Colored two-way arrows denote three different ripple directions which correspond to three different domains. The ripples in one domain are rotated by 60° with respect to those in the neighboring domain. The lower panels show schematic pictures of three-color domain structures (α: gray, β: pink, and γ: sky-blue). Blue and red dots designate the vortex and anti-vortex cores, respectively.

To have a better understanding of the ripple domain structure, we have analyzed the TSM image of the red-dashed rectangular area in Figure 1b. Interesting domain network patterns are observed near the folded edge as shown in Figure 2a. Specifically, we find regularity of ripple domain networks: three distinguishable domains, whose ripple directions have three-fold symmetry, merge at one point (called a core). An even more intriguing observation is that each core can be identified by the vorticity of domain configurations, *i.e.*, as vortex (α–β–γ, clockwise) or anti-vortex (α–β–γ, anti-clockwise). The vortex (blue dot) and the anti-vortex (red dot) are always paired. The nearest neighbor of a vortex along a continuous domain boundary is an anti-vortex and vice versa. Moreover, we can observe this regularity of ripple domain network patterns in other regions of our large monolayer graphene (see Supplementary Figure S5 online).

To investigate the response of ripple domains with external stimuli, we carried out high humidity exposure experiments^24^,^25^. It is known that water can diffuse between graphene and SiO_2_ which often induces lower friction as subsurface lubricant^25^. Such an interaction can induce structural deformation of graphene by introducing wrinkles, folds, and shrinkage. All of these can lead to strain localization and redistribution of stress, resulting in a rearrangement of ripple domain structures. Figure 2b exhibits rearrangement of domain network patterns after one week of exposure of graphene to high humidity (relative humidity of 80–90%). Although high humidity exposure can lead to a change in the domain network patterns of graphene and the interface energy between graphene and SiO_2_/Si substrate due to intercalated water, the regularity of the ripple domain network remains unchanged. It should be noted that the difference between domain contrasts in Figures 2a and 2b is due to different scan directions (see *Methods*).

**Figure 3:**
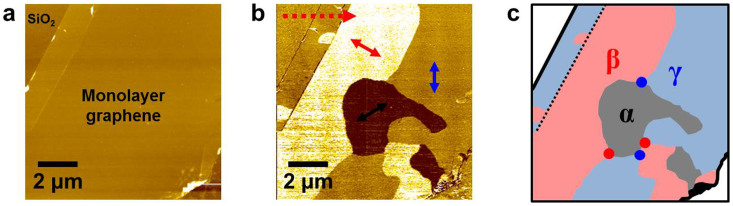
Closed ripple domain surrounded by two pairs of vortex and anti-vortex. (a) AFM topographic image and (b) simultaneously obtained TSM image adjacent to folded and wrinkled edge of monolayer graphene. (c) The corresponding schematic ripple domain structure, where the closed domain is surrounded by two pairs of vortex and anti-vortex.

We now focus on the closed ripple domains that are surrounded by domain boundaries connected to vortices and anti-vortices. It is obvious that there are two γ (Figure 2a) and two β (Figure 2b) closed ripple domains before and after high humidity exposure, respectively. Although the shapes and characteristics of closed ripple domains before and after exposure of graphene to high humidity are different, each closed ripple domain as shown in Figure 2 is surrounded by one pair of vortex and anti-vortex. The closed ripple domains are often observed in the vicinity of folded and wrinkled edges of graphene. The intriguing feature is that the closed ripple domain is usually surrounded by n pairs of vortex and anti-vortex (n = 0, 1, and 2). Figures 2a and 2b show the case of n = 1, whereas Figures 3b and 3c correspond to the case of n = 2. The ripple domain image shown in Figure 3b is obtained from another monolayer graphene sheet with strongly folded and wrinkled edges shown in Figure 3a. Moreover, we can observe a region with only two neighboring domains (n = 0), as seen in Figure 1b, which can be considered as one where strain directions are constrained and cannot cover the directions rotated in the range of −30° to 30° from the positive or negative directions of one zigzag axis.

Vortices that can be considered to be a topological defect are commonly observed in condensed matter, including optical vortex patterns in liquid crystal^26^, vortices in superconductor^27^, multiferroic vortex^20^,^21^, and vortex of ferroelectric domain walls^22^. Such topological defects are controlled by both symmetry breaking and phase transition^18^,^19^,^20^,^21^,^22^,^26^,^27^. The interaction among topological defects can induce the emergence of a vortex and anti-vortex pair at the cost of explicit symmetry breaking^18^,^19^,^20^,^21^. For the topological disorder in polycrystalline graphene grown by chemical vapor deposition, a pair of edge dislocations is observed with the Stone–Wales defect, e.g. a pentagon–heptagon pair, leading to structural deformation by elongation and/or compression of carbon–carbon bonds, shear, and lattice rotations^28^,^29^. In contrast to such surface corrugations arising from topological disorder, the pairing of vortex and anti-vortex in ripple domains of single crystalline exfoliated graphene seems to be unexpected and an intrinsic characteristic of its lattice structure mechanically interacting with surroundings. This is reasonable considering that the ripple directions of three distinctive domains merge at the core having three-fold symmetry. As a result, ripple domain boundaries and their cores can be considered to be new types of topological defects without dislocation because long-range ordered ripples result from the breaking of the lattice symmetry of graphene^30^.

**Figure 4:**
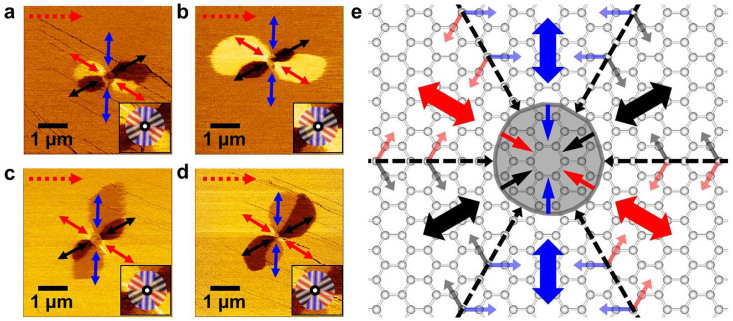
The emergence of radial ripple domain pattern. (a–d) Expanded TSM images showing radial ripple domain patterns, which are obtained by scanning over P1 (a), P2 (b), P3 (c), and P4 (d) in Figure 1a, respectively. Black-, blue-, and red-colored two-way arrows marked on domains represent three-variant directions of ripple. Lower right insets show six-domain configuration around one particle with two sets of three-color domains. (e) Schematic picture of the formation of six ripple domains under radial stress developed by an extraneous particle (shown with shaded area). The development of rippling parallel to the tension (indicated by arrows in black, blue, and red) toward the center and perpendicular to the in-plane compression (indicated by arrows in blurred black, blue, and red) results in the arrangement of six distinct domains. The black-colored dashed arrows represent the expected strain along the armchair direction resulting from radial stress.

Besides the formation of ripple domain networks arising primarily from the surface stress induced by the folds and wrinkles at the edge of the graphene sheet, intriguing radial ripple domain patterns are observed around some particles (Figure 4). During mechanical exfoliation, the particles in question can become sandwiched between the graphene sheet and the SiO_2_/Si substrate. Biaxial stress on graphene can be expected near graphene–particles contact position. We investigated four different regions around the particles, as indicated by red squares in Figure 1a. The radial domain patterns on P1 (Figure 4a) and P2 (Figure 4b) are formed within the region where the ripple direction is indicated by the blue arrow, while the background domain of P3 (Figure 4c) and P4 (Figure 4d) have the ripple direction indicated by the red arrow. As can be seen from Figure 4, each radial domain pattern seems to have a couple of two-lobed shape domains within one background domain. In view of the ripple directions, each pattern has six domains, *i.e.*, two sets of three-color domains (insets of Figures 4a–4d), whose ripples meet at domain boundaries with only 60° forming angle and show six-fold rotational symmetry accompanied by mirror symmetry. We should note that ripple size in the domain around a pariticle is expected to be same as that in the three-fold domain, as observed in Figs. 2 and 3, without any particle because both domains show the same TSM contrast and LFM anisotropy^15^.

The emergence of six ripple domains is a result of the radial stress imposed by the external particles, which is similar to the radial wrinkles in elastic membrane under an axisymmetric traction force at the center^1^,^3^,^4^. It has been suggested that the generated wrinkles are parallel to the applied tension and perpendicular to the applied compression^4^,^5^. On the other hand, in theoretical predictions for elastic properties of graphene nano-ribbons under uniaxial tensile stress, the variation of the bond length and bond angle occurs along or close to the tension direction^31^. It has been also predicted that the variation of C-C bond along the armchair tension direction is much larger than that along the zigzag tension direction^31^, which suggests the easier deformation of C–C bond along the armchair direction. According to the above, six-ripple domain configurations as well as their ripple directions with six-fold rotational symmetry can be understood by the schematic atomic scale drawing shown in Figure 4e. Radial stress around a particle preferentially induces deformation of the C–C bond along armchair directions (black-dashed arrows), leading to in-plane compressive stress in azimuthal directions (blurred black, blue, and red arrows). The azimuthal compressive stress and the remaining radial tensile stress along zigzag directions (black, blue, and red arrows) can cause zigzag directional ripples with six-fold rotational and mirror symmetry. Because graphene has pre-formed ripples along one zigzag direction^17^, the effect of the particle appears strongly along the major zigzag direction and weakly along two other minor zigzag directions, resulting in a couple of two-lobed shape domains within one background domain as shown in Figures 4a–4d. In contrast to an elastic membrane with continuous wrinkling along radial directions, rippling in graphene forms in specific directions that are correlated with its lattice structure.

**Figure 5:**
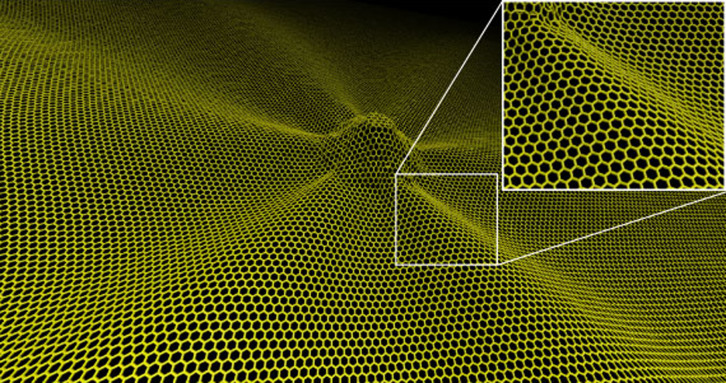
The atomic structure of graphene under a local uplift perpendicularly applied to the plane of graphene obtained by moledular dynamics. The thermal environment was assumed to be at 300 K and the geometry was at the time of 1.6 ns. The inset of the figure is one part of the ripples, which shows that the ripples appear along the zigzag direction.

To indentify the scenario for the six-fold ripple, we performed atomic molecular dynamics on graphene under a local perpendicular uplift. The circular uplift was perpendicularly applied to the graphene and the radius of the protuberant area was ~2.5 nm. We find that the circular uplift induces a six-fold symmetric ripple with respect to the center of the protuberant area and the height of the ripples is ~0.5 nm as shown in Figure 5. In addition, the six-fold ripples appear along zigzag directions, which is consistent with our prediction in Figure 4(e). The out-of plane deformation of the protuberant ares is ~2 nm in high.

## Conclusion

Using the transverse shear microscope (TSM), we observed unique configurations of ripple domains in the exfoliated monolayer graphene where the local stress develops around folded or wrinkled edges and external particles. The redistribution of the strain results in an interesting network of three-color ripple domains with three different ripple directions. Seemingly irregular domain networks exhibit an arrangement of three-color domains meeting at one point (core) and the emergence of vortex and anti-vortex pairing as a new type of topological defect in graphene. In addition, an extraneous particle induces a central force on the surface of graphene, leading to radial stress that results in six-ripple domains around it. The rippling is caused by the coupling between the radial zigzag directional tension and the azimuthal armchair directional compression due to the unique features of a graphene lattice, which is supported by theoretical simulation using molecular dynamics. Our observation provides an essential understanding of ripple physics for application of graphene in flexible and nanoscale devices.

## Methods

### Preparation of graphene

Mechanical exfoliation method was used to prepare graphene sheets on the SiO_2_ (300 nm)/Si substrate under ambient condition. Using an optical microscope, we observed the exfoliated graphene sheets of various sizes and shapes.

### Characterization of graphene

We obtained AFM topography, LFM, and TSM images simultaneously with the contact mode using two AFMs (SPA-300HV from SEIKO and XE-100 from PSIA) at ambient condition. Because scan size of PSIA XE-100 AFM (50 μm) is much larger than that of SEIKO SPA-300HV AFM (20 μm), PSIA XE-100 AFM has the advantage for observing a large area of the graphene sheet, as shown in Figure 1. As PSIA XE-100 has a low resolution, we used SEIKO SPA-300HV AFM to obtain high resolution TSM images at ambient condition. We used lateral friction tips (PPP-LFMR with spring constant of 0.2 N/m, Nanosensors) to obtain AFM topographic and TSM images. During AFM measurements, we maintained the loading force at 0 nN. The definition of AFM scan directions has been described in earlier publications^15^,^16^. In order to obtain TSM and LFM images to determine ripple directions, we need two different scan directions: one being a lateral scan when the AFM cantilever is moved perpendicular to the cantilever arm, and the other being a longitudinal scan when the AFM cantilever is moved parallel to the cantilever arm. Since the TSM provides much clearer image for observing the configuration of ripple domain structures than LFM^16^, we can investigate the ripple domain structures in various exfoliated monolayer graphene sheets using TSM images.

### Theoretical simulation

We performed atomic molecular dynamics on graphene using the consistent valence forcefield (CVFF)^32^, which includes the bond-stretching energy term, the angle-bending energy term, the torsion-angle (dihedral) energy term, the out-of-plane energy term (improper torsion), and the van der Waals energy term. In the CVFF, the bond-stretching and angle-bending energy terms were described by a simple harmonic potential form, and the van der Waals energy term was described by the Lennard-Jones (12-6) potential form. The large-scale atomic molecular massively parallel simulator (LAMMPS) package^33^ was used for all simulations. For the molecular dynamics simuations, 70 nm × 70 nm graphene with 186,304 carbon atoms was used. The Nose-Hoover thermostat was used and the thermal environment is 300 K.

## Author Contributions

J.S.C., T.C. and B.H.P. planned the experiments; Y.P., M.J.L. and J.S.C. prepared samples; Y.P. carried out AFM measurements with the help of J.S.C. and M.J.L.; M.P. and H.L. performed and analyzed the calculations; Y.P., J.S.C., T.C., Q.J. and B.H.P. interpreted the results; all authors participated in discussion and writing the manuscript.

## Supplementary Material

Supplementary Information Supplementary Information
