# Supplementary material for: Configuration of ripple domains and their topological defects formed under local mechanical stress on hexagonal monolayer graphene
Source: Sci Rep. 2015 Mar 24;5:9390. doi: 10.1038/srep09390 (PMC4371081; doi:10.1038/srep09390)
Supplement: Supplementary Information [file srep09390-s1.doc]

Supporting Information

**Configuration of ripple domains and their topological defects formed under local mechanical stress on hexagonal monolayer graphene**

**Yeonggu Park^1†^, Jin Sik Choi^2†^, Taekjib Choi^3†^, Mi Jung Lee^1^, Quanxi Jia^1, 4^, Minwoo Park^1^, Hoonkyung Lee^1^, and Bae Ho Park^1*^**

^1^ Division of Quantum Phases & devices, Department of Physics, Konkuk University, Seoul, 143-701, Korea

^2^ Creative Research Center for Graphene Electronics, Electronics and Telecommunications Research Institute (ETRI), Daejeon 305-700, Korea

^3^ Hybrid Materials Research Center, Department of Nanotechnology and Advanced Materials Engineering, Sejong University, Seoul 143-747, Korea

^4^ Center for Integrated Nanotechnologies (CINT), Los Alamos National Laboratory, Los Alamos, New Mexico 87545, USA

^†^These authors equally contributed to this work.

*Correspondence and requests for materials should be addressed to B. H. P. (email: baehpark@konkuk.ac.kr)


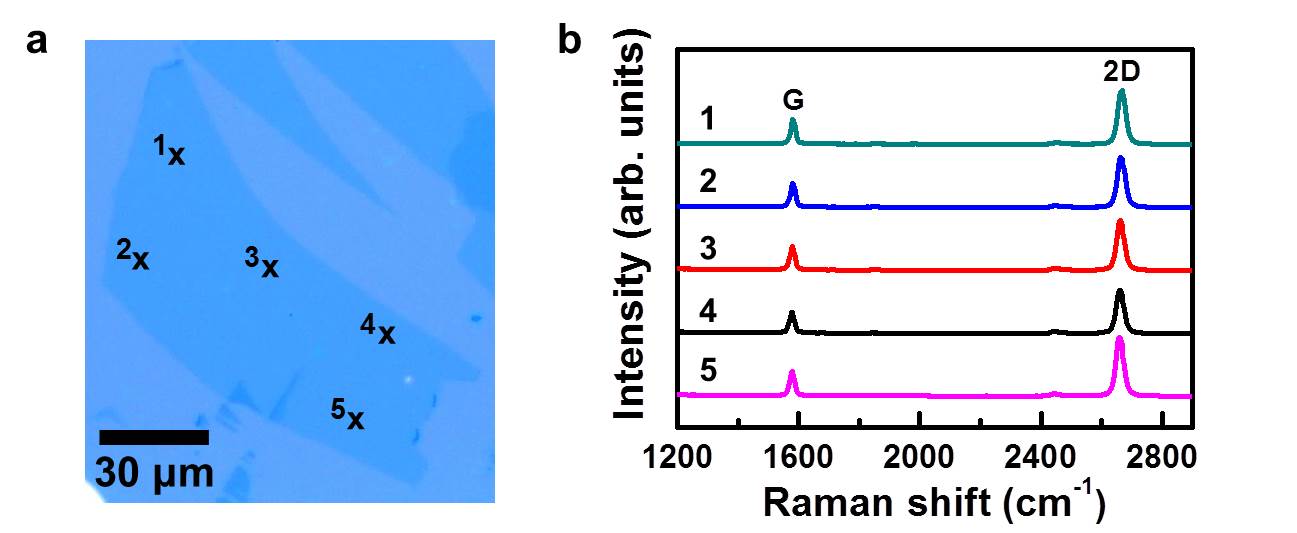


**Figure S1.** (a) Optical microscopic image of exfoliated monolayer graphene. (b) Raman spectra at five arbitrary points, except for structural deformations such as folded and wrinkled edges.

The exfoliated graphene sheet was identified by optical microscope as shown in Figure S1a. To confirm the number of exfoliated graphene layers, we carried out micro-Raman spectroscopy at five arbitrary points at the middle of the graphene sheet, excluding folded and wrinkled edges. The resultant spectra show typical features of exfoliated monolayer graphene on SiO_2_ substrate^1,2^: G and 2D peaks at ~1580 cm^-1^ and ~2680 cm^-1^, respectively; relatively high intensity ratio of 2D peak to G peak (I_2D_/I_G_); sharp and symmetric 2D peak with a single Lorentzian line; absence of D peak at ~1350 cm^-1^, associated with structural defects in the graphene sheet. Moreover, we cannot observe significant differences in Raman spectra measured at the five different locations, which indicates that the monolayer graphene does not contain charge doping variations or high-strain.


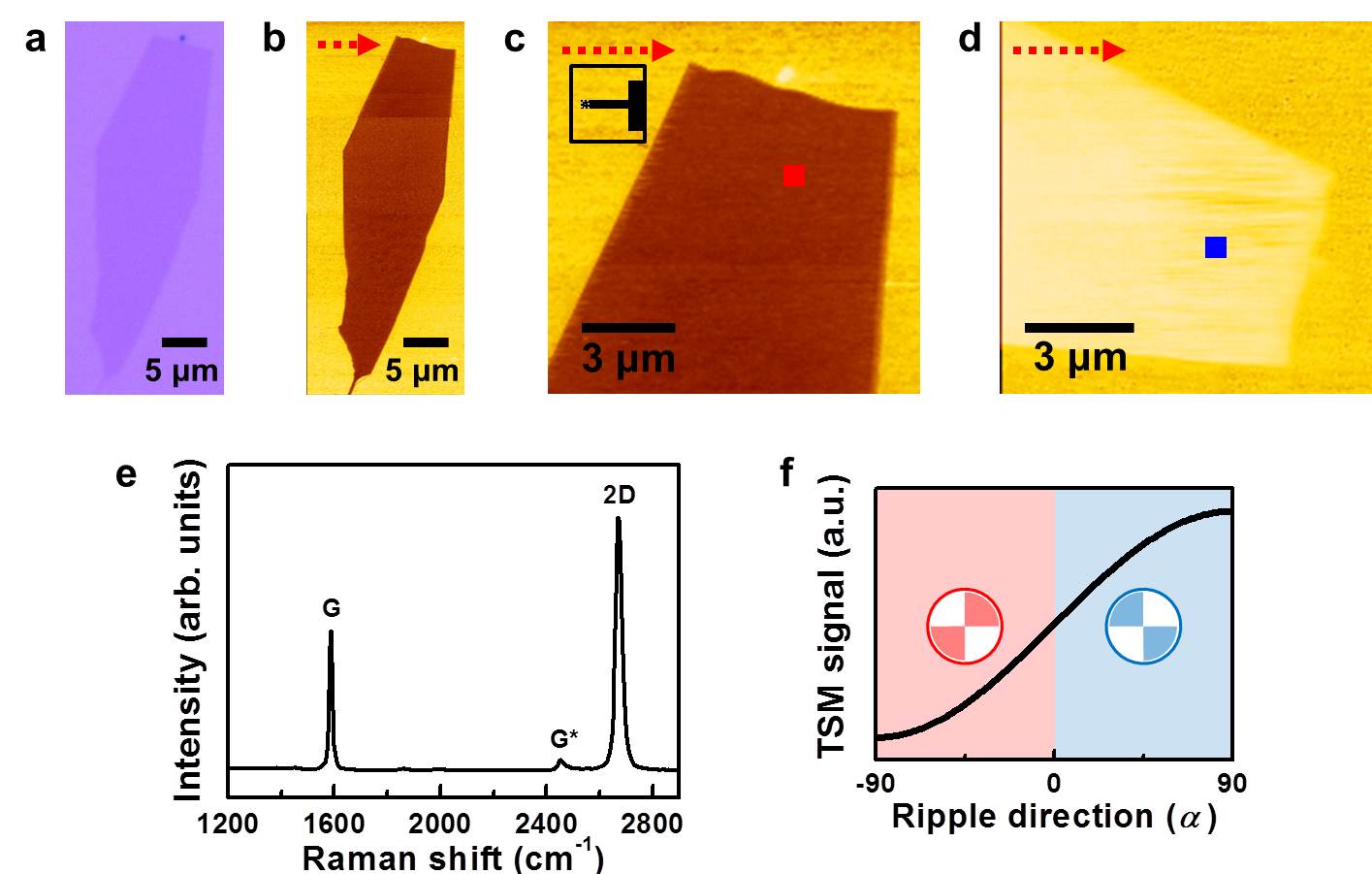


**Figure S2.** (a, b) Optical microscopic (a) and TSM (b) images of an exfoliated monolayer graphene with single ripple domain. (c, d) Expanded TSM images of initial (c) and 90°-clockwise-rotated (d) graphene. The inset of (c) shows the schematic top-view image of the AFM cantilever. The red dashed line denotes the scan direction of AFM tip during obtaining TSM image. (e) Raman spectrum measured at the middle of the monolayer graphene sheet. (f) Calculated ripple direction dependence of TSM signal. The inset shows the possible ripple direction ranges of the graphene single domains designated by red and blue colors, which are estimated from TSM images of Figures S2c and S2d, respectively.

We find a single ripple domain structure in the whole exfoliated graphene sheet. We cannot see any contrast distributions in both optical microscopic and TSM images of the exfoliated monolayer graphene sheet as shown in Figures S2a and S2b, respectively. We note that wrinkled and folded edges and/or external particles are not observed, which can cause local stress distribution in the monolayer graphene sheet. Figure S2c is the expanded TSM image of the upper area in Figure S2b. When the sample is rotated 90° clockwise, the relative contrast of the graphene in TSM image is changed from dark to bright as shown in Figure S2d. Previously we found that, by considering the contrast of TSM image, we can determine whether the ripple is rotated in the clockwise or counterclockwise direction from the cantilever body axis (inset of Figure S2c)^3^. Since bulk SiO_2_ gives rise to isotropic TSM images^3^, these anisotropic TSM images may result from linearly aligned ripples in graphene. The calculated ripple direction dependence of TSM signal follows sin*α* function as shown in Figure S2f, where *α* (-90° < *α* < 90°) is the rotation angle of the ripple (counter-clockwise) from the lateral direction of the cantilever body^3^. Because the single domains in Figures S2c and S2d show dark and bright TSM image contrasts, respectively, we can determine the possible ripple directions of the domains denoted by red and blue colors, as -90° < α_red_ < 0° and 0° < α_blue_ < 90° (inset of Figure S2f).


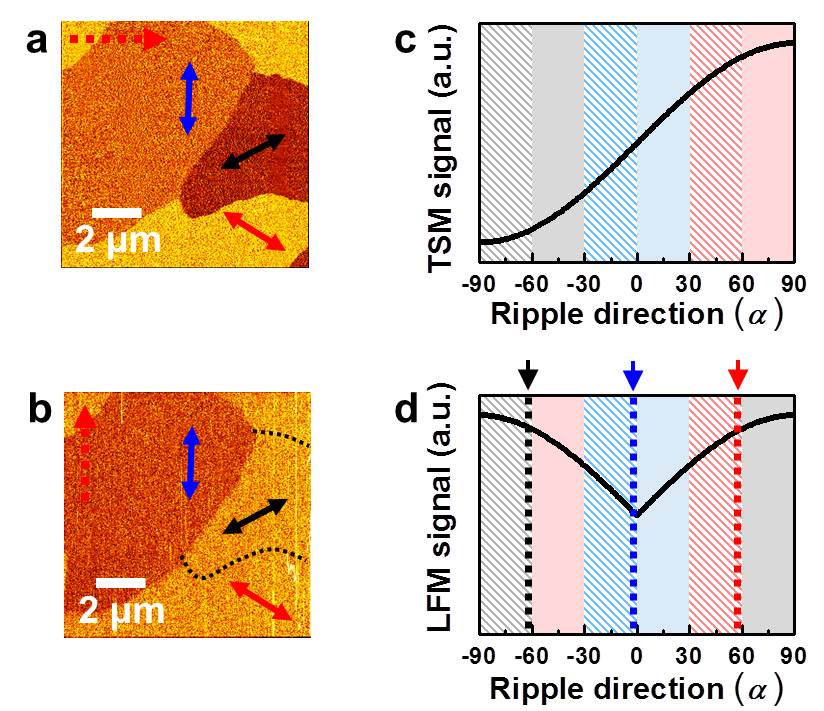


**Figure S3.** (a, b) TSM (a) and LFM (b) images for an exfoliated monolayer graphene with three-color ripple domains. Resultant ripple directions in both images are expressed by three different colored two-way arrows. Black dotted curves in Figure S3b refer to boundaries between ripple domains with similar contrasts but different ripple directions. (c, d) Calculated ripple direction (*α*) dependence of TSM (c) and LFM (d) signals. Colored area or dotted line in Figures S3c and S3d indicate the ripple direction of the same colored domain, which is estimated from the contrast in TSM and LFM images.

We chose one region of an exfoliated monolayer graphene containing three-color ripple domains and obtained TSM and LFM images during longitudinal and lateral scans as shown in Figures S3a and S3b, respectively. In order to determine each ripple direction, we used the previously reported facile method comparing contrasts between TSM and LFM images^3^. TSM and LFM signals follow sinα and |sin*α*| functions as shown in Figures S3c and S3d, respectively^3,4^. Considering that ripple direction of one domain is rotated by 60° or -60° from that of an adjacent domain, we can estimate the ripple direction of each domain within a range of 60° from both image. The bright-, medium-, and dark-contrast domains in a TSM image (Figure S3a), which are denoted by red, blue, and black colors, have α values in the range of 30° to 90°, -30° to 30°, and -90° to -30°, respectively (Figure S3c). On the other hand, the contrast of LFM image (Figure S3b) implies that the red-, blue-, and black-colored domains should have -60° < α_red_ < -30° or 30° < α_red_ < 60°, -30° < α_blue_ < 30°, and -90° < α_black_ < -60° or 60° < α_black_ < 90° (Figure S3d). By comparing α values estimated from TSM and LFM images, we can assign ripple directions for each domain within a range of 30°: 30° < α_red_ < 60°; -30° < α_blue_ < 0°; -90° < α_black_ < -60°. Moreover, observing almost the same LFM image contrast of black- and red-colored domains, we can determine the precise ripple direction of each domain: α_black_ = -62°; α_blue_ = -2°; α_red_ = 58°; as denoted by the colored two-way arrows in Figures S3a and S3b.


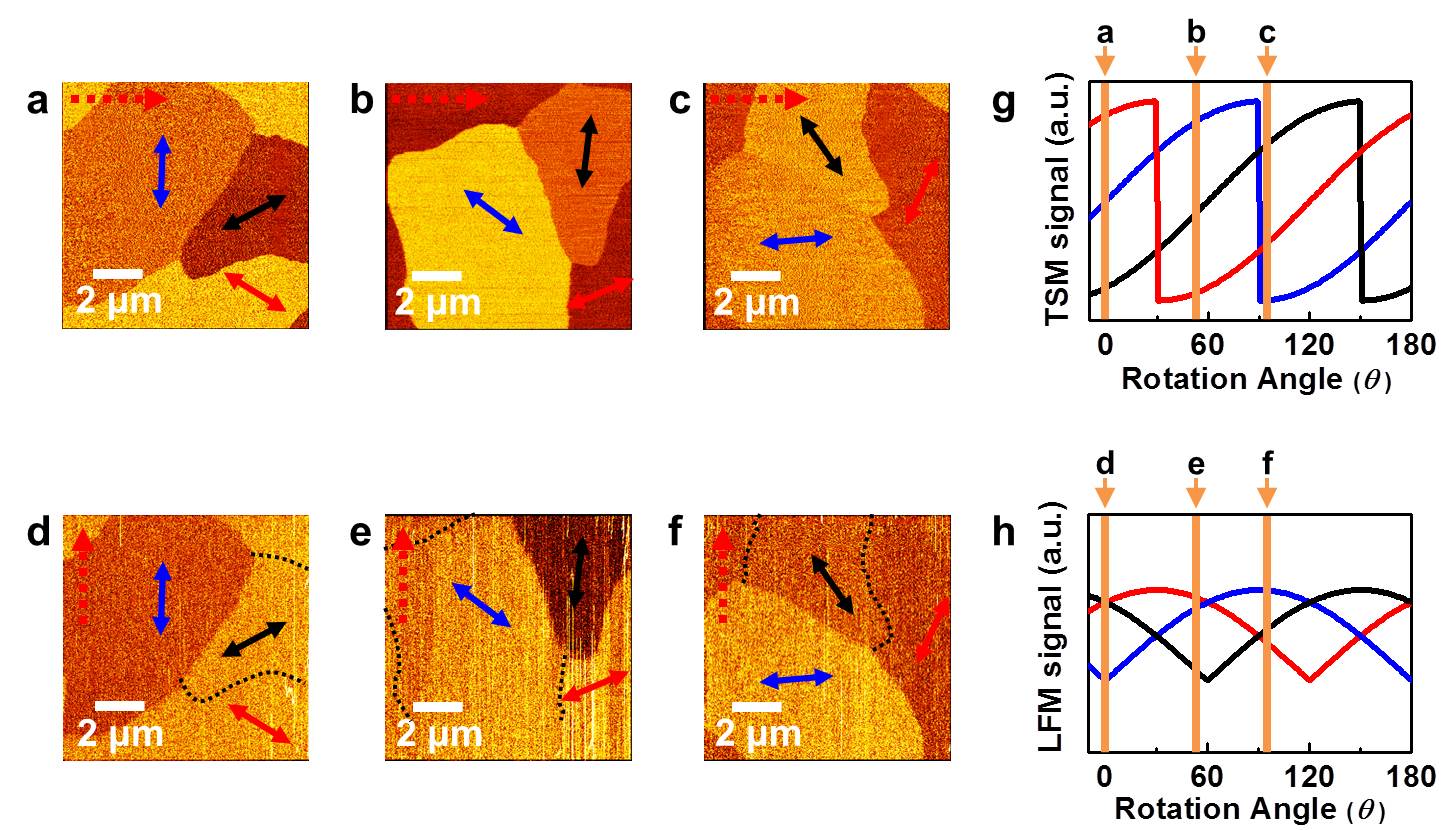


**Figure S4.** (a–f) TSM (a-c) and LFM (d-f) images of 0° (a, d), 55° anti-clockwise (b, e), and 97° anti-clockwise (c, f) rotated monolayer graphene. Each ripple direction is expressed by a colored two-way arrow. Black dotted curves in Figures S4d–S4f refer to boundaries between ripple domains with similar contrasts but different ripple directions. (g, h) Expected sample rotation angle (*θ*) dependences of TSM (g) and LFM (h) signals of domains with ripple directions designated by red-, blue-, and black-colored two-way arrows in Figures S4a–S4f. Orange-colored vertical lines denote sample rotation angles corresponding to those of images in Figures S4a–S4f.

Previously, we have observed that each ripple domain shows sample rotation angle dependent TSM and LFM signal with a period of 180° and 60° shifted with respect to that of a neighboring domain^3,4^, which can be explained by the linearly aligned ripple in each domain. We performed the sample rotation experiment to confirm whether the ripple directions estimated from both images in Figures S3a and S3b can explain the experimental data. We obtained the TSM and LFM images for three arbitrary sample rotation angles (0°, 55° anti-clockwise and 97° anti-clockwise). Figures S4a–S4f show that contrasts of TSM and LFM images change as the sample is rotated. On the other hand, the estimated ripple direction of each domain can provide us the expected sample rotation angle (*θ*) dependence of TSM and LFM signal in each domain, as shown in Figures S4g and S4h, respectively. Orange-colored vertical lines denote sample rotation angles corresponding to those of Figures S4a–S4f. We note that the contrasts of expected TSM and LFM signals of three-color domains in one orange-colored vertical line of Figures S4g and S4h are in good agreement with those of the experimentally obtained corresponding TSM and LFM images in Figures S4a–S4f. Furthermore, we confirm that ripple directions determined from TSM and LFM images are comparable to those obtained from a circle with 60° fan shape sectors indicating the ripple domain distribution around a particle, as shown in the insets of Figures 4a–4d.


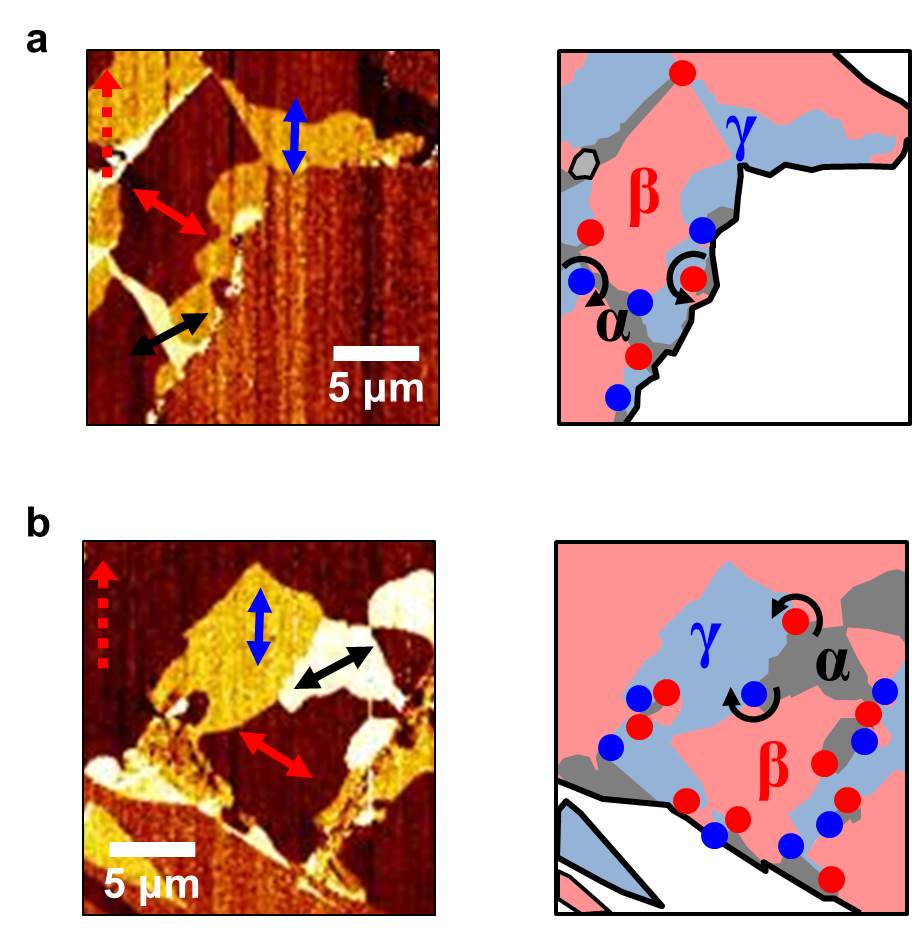


**Figure S5.** (a b) Magnified TSM images (left panels) and schematic pictures (right panels) of three-color domain structures (α: gray, β: pink, and γ: sky-blue) in different regions of the large monolayer graphene in Figure 1b. Colored two-way arrows denote three different ripple directions which are characteristics of three different domains. Blue and red dots designate the vortex and anti-vortex cores, respectively, which have clockwise and anti-clockwise vorticity of α, β, and γ domains.

We can find the three-color domain network patterns in magnified TSM images obtained at different regions of the large monolayer graphene shown in Figure 1b. We can reconfirm the regularity of ripple domain boundary in these two complicated domain configurations: existence of core which is a point merging three-color ripple domains; identification of core type by the vorticity of domain configuration; vortex and anti-vortex pairing.

**References:**

1. Ferrari, A. C. *et al.* Raman spectrum of graphene and graphene layers. *Phys. Rev. Lett.* **97**,187401 (2006).

2. Ferrari, A. C. & Basko, D. M. Raman spectroscopy as a versatile tool for studying the properties of graphene. *Nat. Nanotech.* **8**, 235– 246 (2013).

3. Choi, J. S. *et al.* Facile characterization of ripple domains on exfoliated graphene. *Rev. Sci. Instrum.* **83**, 073905 (2012).

4. Choi, J. S. *et al.* Friction anisotropy–driven domain imaging on exfoliated monolayer graphene. *Science* **333**, 607–610 (2011).
